# Expression and function of the TL1A/DR3 axis in chronic lymphocytic leukemia

**DOI:** 10.18632/oncotarget.5201

**Published:** 2015-09-15

**Authors:** Chiara Cavallini, Ornella Lovato, Anna Bertolaso, Elisa Zoratti, Giorgio Malpeli, Elda Mimiola, Martina Tinelli, Fiorenza Aprili, Cristina Tecchio, Omar Perbellini, Aldo Scarpa, Alberto Zamò, Marco Antonio Cassatella, Giovanni Pizzolo, Maria Teresa Scupoli

**Affiliations:** ^1^ Interdepartmental Laboratory of Medical Research (LURM), University of Verona, Verona, Italy; ^2^ Department of Pathology and Diagnostics, Section of Pathological Anatomy, University of Verona, Verona, Italy; ^3^ Applied Research on Cancer-Network (ARC-NET), University of Verona, Verona, Italy; ^4^ Department of Medicine, Section of Hematology, University of Verona, Verona, Italy; ^5^ Department of Pathology and Diagnostics, Laboratory of Cytogenetics, Azienda Ospedaliera Universitaria Integrata Verona, Verona, Italy; ^6^ Department of Pathology and Diagnostics, Section of General Pathology, University of Verona, Verona, Italy

**Keywords:** leukemia, microenvironment, chronic lymphocytic leukemia, cytokines, cytokine receptors

## Abstract

TNF-like ligand 1A (TL1A) and its unique receptor death receptor 3 (DR3) acts as broad T-cell costimulator involved in regulatory mechanisms of adaptive immune response under physiological and pathological settings. Moreover, we have recently shown that TL1A negatively regulates B-cell proliferation. Despite increasing interest on the TL1A/DR3-axis functions, very little is known on its expression and role in leukemia. In this study, we investigated the expression and function of TL1A/DR3 axis in chronic lymphocytic leukemia (CLL). DR3 was differentially expressed in activated CLL cells and predominantly detected in patients with early clinical stage disease. Soluble TL1A has been revealed in the sera of CLL patients where higher TL1A levels were associated with early stage disease. T cells, monocytes and leukemic B cells have been identified as major sources of TL1A in CLL. The relevance of these findings has been sustained by functional data showing that exogenous TL1A reduces CLL proliferation induced by stimulation of the B cell receptor. Overall, these data document the expression of the TL1A/DR3 axis in early-stage CLL. They also identify a novel function for TL1A as a negative regulator of leukemic cell proliferation that may influence the CLL physiopathology and clinical outcome at an early-stage disease.

## INTRODUCTION

Death receptor 3 (DR3/TNFRSF25) is a member of the TNFR superfamily that contains a death domain (DD) as part of its intracellular domain [1–3]. DR3 expression is restricted to lymphocyte-enriched tissues, including peripheral blood leukocytes, thymus and spleen [4]. It is especially up-regulated in activated T cells [4, 5]. Moreover, we have recently reported that it is also expressed on activated B cells [6]. The unique ligand for DR3 is TNF-like ligand 1A (TL1A/TNFSF15), a member of the TNF superfamily [7–9]. TL1A is expressed in a variety of cell types, including activated endothelial cells, monocytes, macrophages, dendritic cells, and T cells [7, 10–13]. Ligation of DR3 with TL1A induces costimulation of T cells [5, 7, 11, 14] and the TL1A/DR3 axis is implicated in regulatory mechanisms of adaptive immune response under physiological and pathological settings [15]. In contrast with this activating function, we have recently showed that TL1A negatively regulates proliferation of B cells activated by BCR stimulation and IL-2 [6].

Chronic lymphocytic leukemia (CLL) patients exhibit a variable clinical course, with some patients having indolent disease and others experiencing a more accelerated course, treatment resistance and dismal outcome [16, 17]. Although the reason for this outcome disparity is not fully understood, the variable clinical course of CLL reflects biological differences that are driven by intrinsic defects as well as external cues deriving from the microenvironment [18–20]. Both cell-intrinsic and microenvironment-mediated events converge on the activation of regulatory signaling pathways involved in processes promoting resistance to apoptosis and overturning the control of cell proliferation [18–20]. Indeed, although CLL has long been regarded as a disease of accumulation rather than proliferation, evidence from *in vivo* labeling experiments [21] and analysis of telomeres [22] has revealed that, in lymphoid tissues, CLL cells proliferate at a relatively high rate. Among the microenvironmental stimuli that may induce CLL proliferation, a fundamental role is played by the B-cell receptor (BCR) signaling, which also represents the most prominent pathogenic mechanism in CLL [23–25].

The proliferation rate is associated with disease activity and progression [21]. Therefore, molecular mechanisms altering the balance between cell proliferation and death in disfavor of cell proliferation may result in clearance of leukemic cells and influence the pathogenic process and clinical outcome. However, little is known to date on molecular mechanisms involved in negative regulation of CLL proliferation.

In this study, we report that CLL cells activated by BCR stimulation differentially express DR3 molecules, which is more frequently associated with early-stage disease. Consistently, soluble TL1A has been detected in sera of CLL patients with an early-stage disease. Moreover, we show that in CLL TL1A is produced by T cells, monocytes and leukemic B cells. Stimulation of DR3 with exogenous TL1A reduces CLL proliferation mediated by the BCR stimulation. Taken together, these results suggest that the molecular axis TL1A/DR3 is a feature of CLL early-stage disease and may play an important role in controlling the proliferation of leukemic cells.

## RESULTS

### DR3 is differentially expressed in activated CLL cells and relates to disease stage

**Figure 1:**
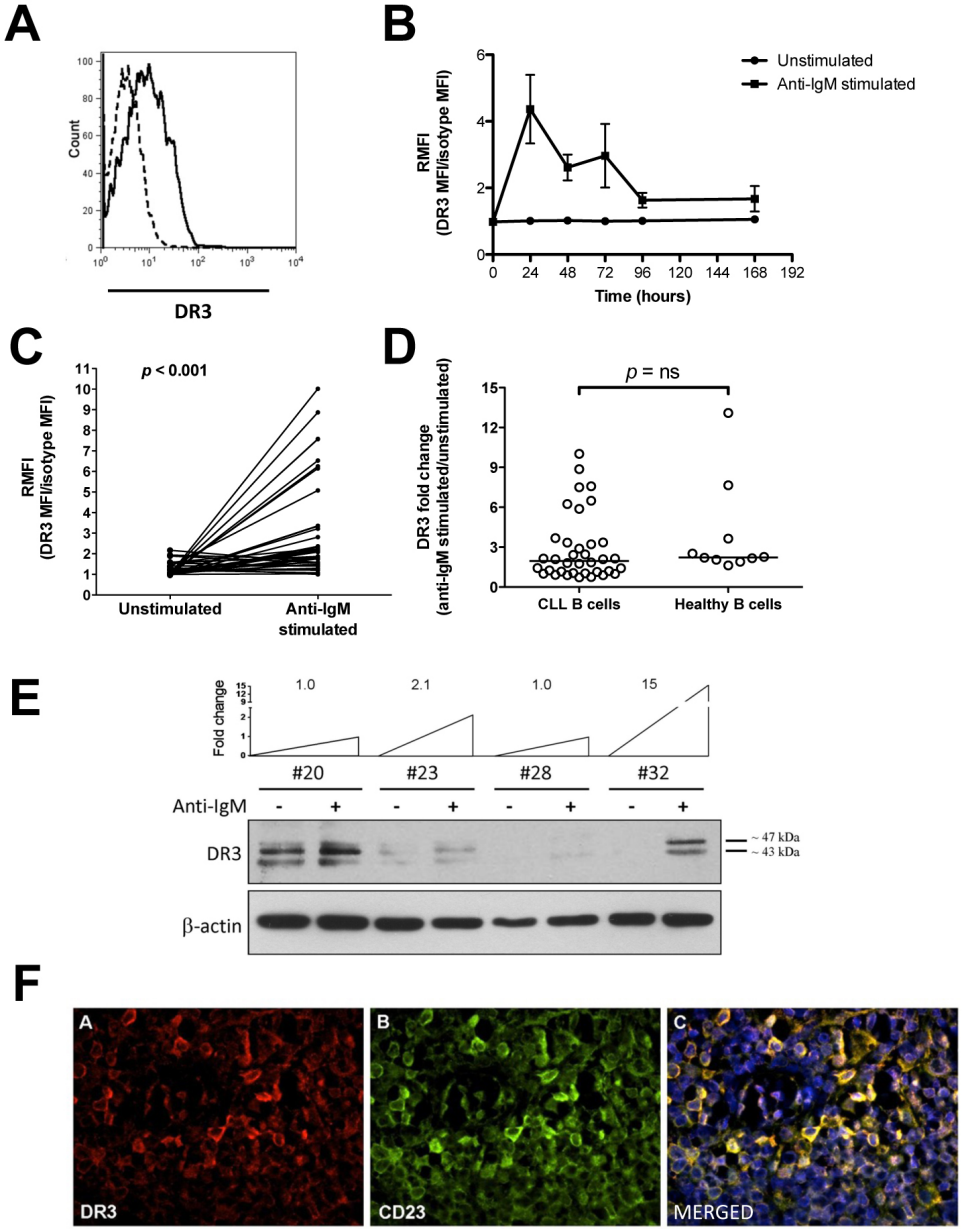
DR3 surface expression in CLL cells **A.** Representative example of DR3 flow cytometry analysis. Dashed line: isotypic control; regular line: anti-DR3 signal. **B.** Time course analysis of DR3 expression in unstimulated and anti-IgM-stimulated CLL cells (*n* = 8). **C.** Surface DR3 expression in unstimulated and anti-IgM-stimulated CLL cells (*n* = 36). Data are expressed as DR3-expression median fluorescence intensity (MFI) divided by isotype-matched control (relative median fluorescence intensity = RMFI). Comparison between unstimulated and anti-IgM-stimulated CLL cells was performed by the two-sample Wilcoxon signed rank sum test. **D.** Comparison of DR3 expression between B cells from CLL samples (*n* = 36) and age-matched healthy donors (*n* = 10). Lines represent median values. Comparison was performed using the Mann-Whitney test. ns = not significant. **E.** Western blot analysis of cell lysates from purified CLL cells (*n* = 4), in unstimulated and anti-IgM stimulated conditions. Level of DR3 induction after anti-IgM stimulation is reported as fold change. **F.** Representative example of DR3 immunofluorescence analysis in CLL lymph-node tissue sections (*n* = 2). Panel A: pseudocolour image of DR3 (200×); Panel B: pseudocolour image of CD23 (200×); Panel C: merged pseudocolour image of CD23 (green), DR3 (red) and DNA (blu) (200×).

DR3 expression was analyzed in CLL cells under basal (unstimulated) conditions and following BCR stimulation (anti-IgM-stimulated), at different time points. Under basal conditions, CLL cells expressed low levels of DR3 when they were either freshly isolated (median RMFI = 1.00, range = 1.00–1.99 RMFI, *n* = 35; data not shown) or cultured in the absence of exogenous stimuli (Figures 1A–1C). Following BCR stimulation, a fraction of leukemic cells expressed increased levels of surface DR3 (Figures 1A–1C) that was sustained for 72 hours in culture and maximal at 24 hours (Figure 1B). Therefore, all subsequent analyses were performed at the 24-hour time point. As shown in Figure 1C, stimulation of the BCR for 24 hours induced a statistically significant increase of DR3 expression in CLL cells (*p* < 0.001), with great variability amongst leukemic cell samples [variance (σ^2^) = 6.33]. Comparison of anti-IgM-induced DR3 expression in leukemic and healthy-donor B cells (σ^2^ = 13.5) revealed no significant differences (Figure 1D). Flow cytometry data were then confirmed by Western blot analysis. DR3 exists as at least 11 isoforms generated by pre-mRNA alternative splicing. The major isoform has a molecular weight of 47 kD [26]. Accordingly, several isoforms were identified in CLL lysates by antibody directed towards the intracellular domain of DR3 (Figure 1E). Those included the isoform at 47 kD and an isoform at approximately 40 kD (Figure 1E). Consistent with flow cytometry data, BCR-stimulation increased or *de novo* induced all the two DR3 isoforms in some CLL cell samples (Figure 1E). To confirm the relevance of our findings, we analyzed DR3 expression in lymph-node specimens from CLL patients using a three-color immunofluorescence approach. Figure 1F (panel A) shows that DR3 is expressed by many cells within the CLL lymph nodes. Panel B shows that many of the cells in this area express CD23. The merged figure in panel C (yellow) shows that DR3-positive cells also express CD23, suggesting that they may include CLL B cells.

**Figure 2:**
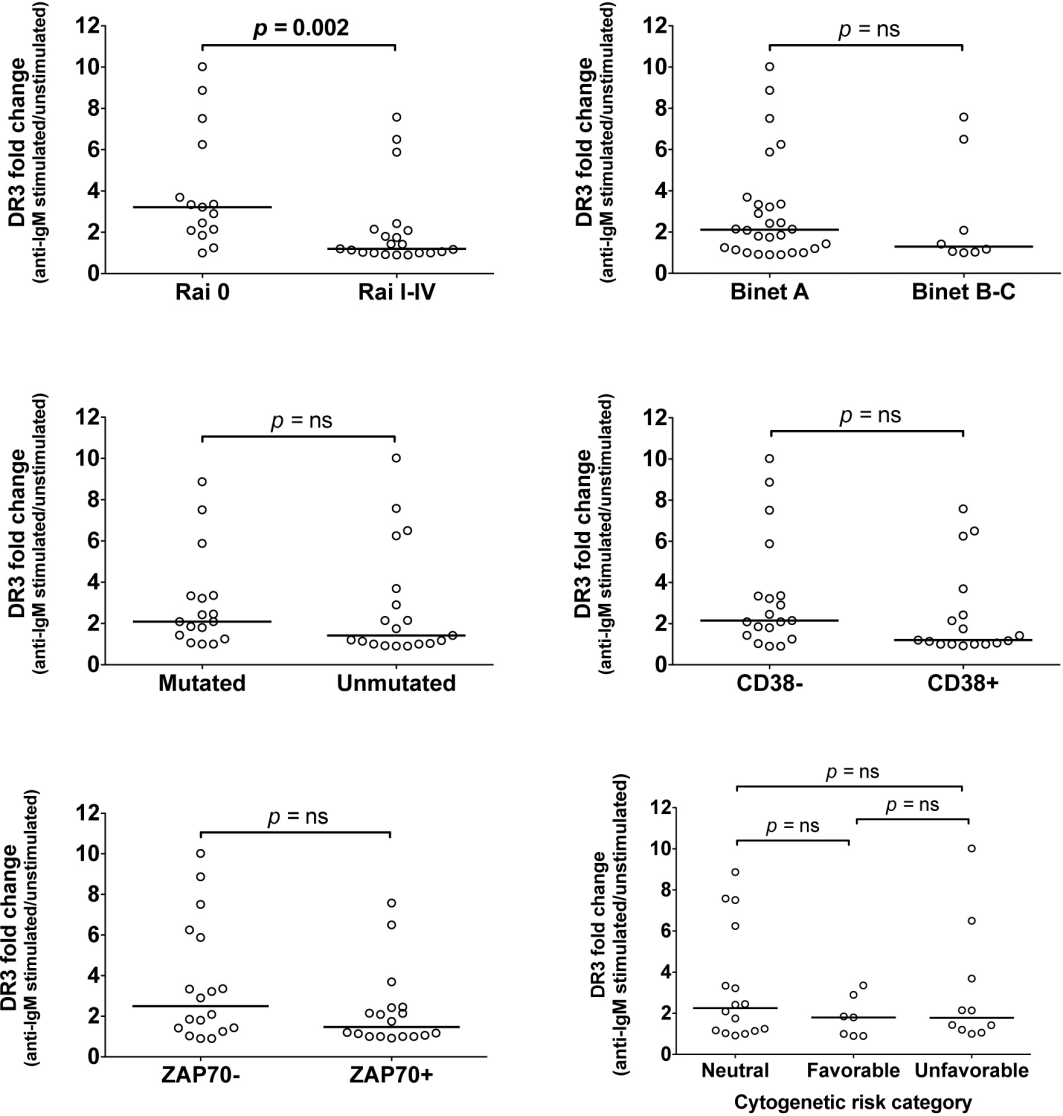
Association of DR3 expression with prognostic parameters DR3 expression was associated with clinical staging according to Rai or Binet classification, or with standard prognostic markers: *IGHV* status, CD38 and ZAP-70 expression, and cytogenetic risk categories. Lines represent median values. Comparison was performed using the Mann-Whitney test and Kruskal-Wallis test. ns = not significant.

To assess whether DR3 could be differentially expressed in prognostic classes of CLL patients, induced DR3 expression was evaluated in relation to clinical staging (according to Rai or Binet classification), standard molecular prognostic markers (*IGHV*, CD38 or ZAP-70 status), or cytogenetics. Mann-Whytney test showed statistically significant concordance between higher induced DR3 expression and Rai 0 stage (Figure 2). In contrast, no significant association was detected between DR3 expression and Binet staging, the status of standard prognostic markers (*IGHV*, CD38, ZAP-70), or cytogenetics (Figure 2). Overall, these results show that DR3 expression identifies CLL patients at an early clinical stage disease and prompted us to detect the presence of TL1A in CLL.

### TL1A is detected in CLL serum and is associated with early-stage disease and absence of CD38

**Figure 3:**
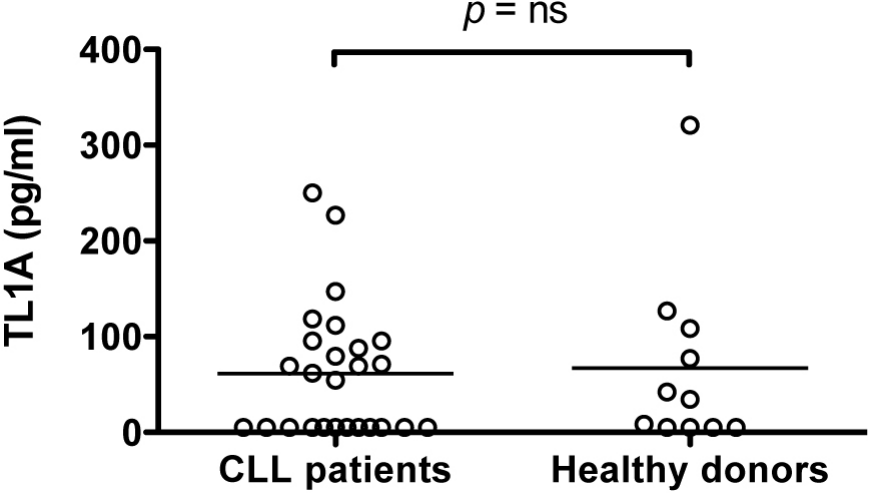
Serum levels of TL1A in CLL patients and healthy donors TL1A serum levels in 26 CLL samples and 11 age-matched healthy donors were measured by ELISA assay. Lines represent median values. Comparison was performed using the Mann-Whitney test. ns = not significant.

Like other TNF members, TL1A contains a predicted transmembrane domain and a bioactive, proteolytically cleaved truncated form that can be released as a soluble factor [8]. The presence of DR3 in CLL cells, led us to hypothesize that a soluble form of TL1A might be detected in the sera of CLL patients. Therefore, serum concentrations of TL1A were measured in patients for whom serum samples were available and in age-matched healthy donors. Serum levels of soluble TL1A were detected in a fraction of CLL patients (14/26; 54%; σ^2^ = 9036) and healthy donors (6/11; 54%; σ^2^ = 4721). No significant differences in TL1A concentrations were revealed between the two groups (Figure 3).

**Figure 4:**
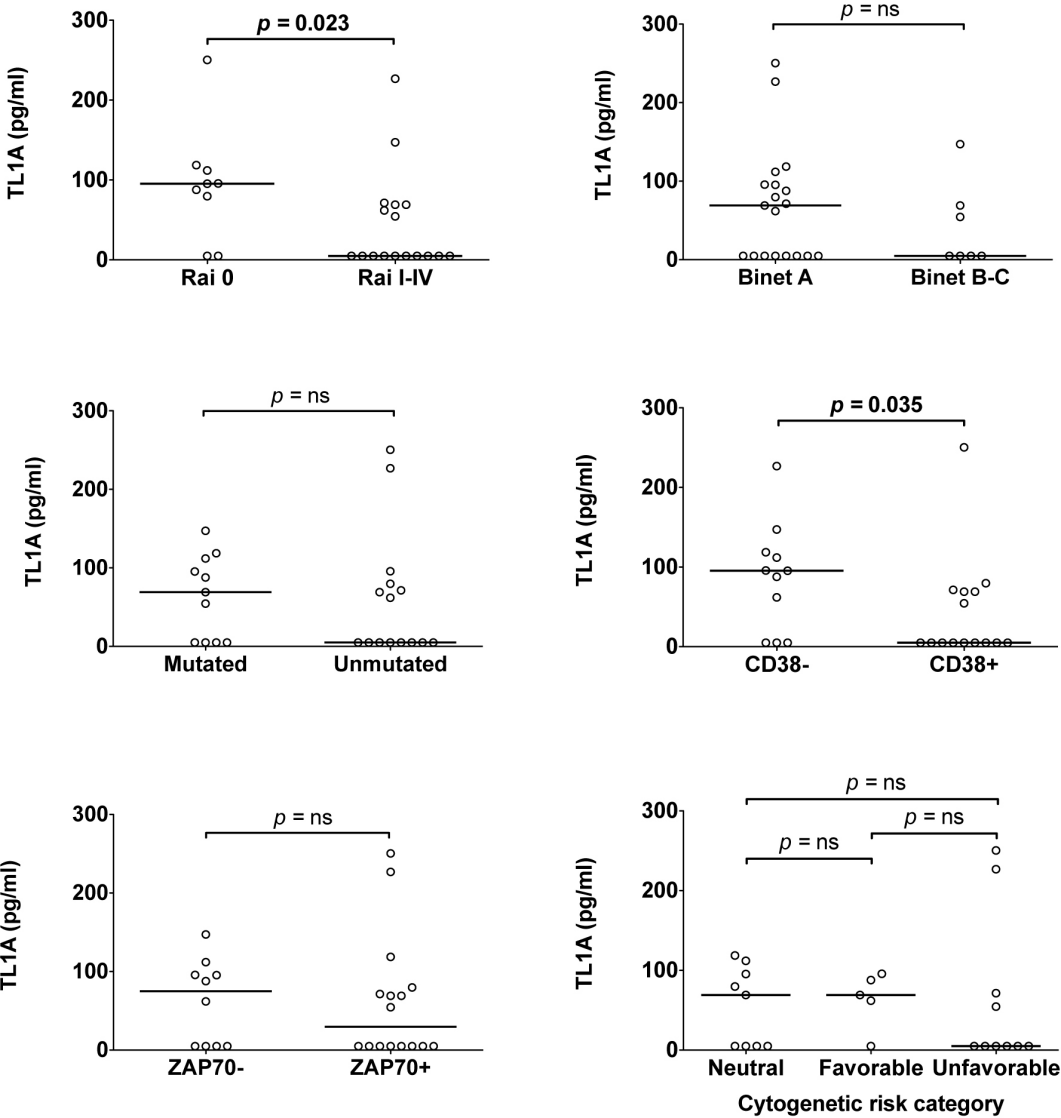
Association of TL1A serum levels with prognostic parameters TL1A serum level was associated with clinical staging according to Rai or Binet classification, or with standard prognostic markers: *IGHV* status, CD38 or ZAP-70 expression, cytogenetic risk categories. Lines represent median values. Comparison was performed using the Mann-Whitney test and Kruskal-Wallis test. ns = not significant.

To explore the pathophysiological impact of TL1A in CLL, serum TL1A concentrations were related to clinical staging (according to Rai or Binet classification), the prognostic marker status (*IGHV*, CD38 or ZAP-70), or cytogenetics (Figure 4). Remarkably, Mann-Whytney test showed statistically significant concordance between higher TL1A serum concentration and Rai 0 stage or absence of CD38 expression. In contrast, differences of TL1A concentrations across CLL prognostic classes defined by Binet clinical staging, *IGHV* status, ZAP-70 expression, or cytogenetics were not statistically significant, although TL1A levels were tendentiously higher in patients at a Binet A stage, with mutated *IGHV*, ZAP-70 negative, or neutral/favorable cytogenetics. Overall, these data document the presence of TL1A in sera of CLL patients with an early clinical stage disease and prompted us to search for the cell source of TL1A in CLL.

### TL1A is expressed by PBMC cell subsets from CLL and healthy donors

**Figure 5:**
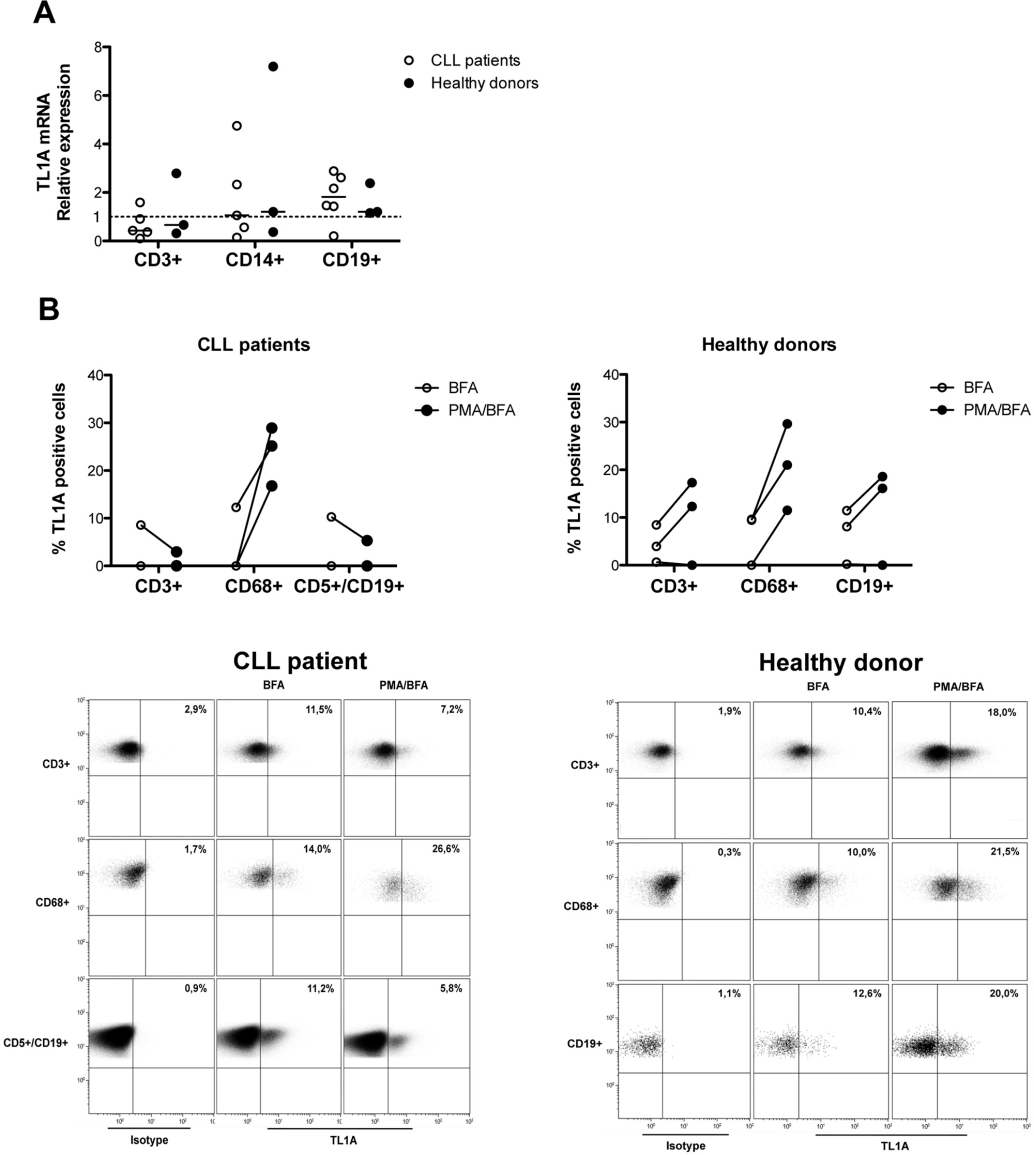
TL1A expression in PBMC cell subsets from CLL patients and healthy donors **A.** Quantitative real-time PCR analysis of TL1A mRNA expression in FACS-isolated cell subsets (CD3^+^, CD14^+^, and CD19^+^) from CLL (*n* = 6) and healthy donors (*n* = 3). Gene expression was normalized *vs* β-actin expression and represented as relative expression of TL1A mRNA levels with respect to mean TL1A mRNA across samples (set as 1). Lines represent median values. **B.** TL1A protein expression analysis in gated-cell subsets (CD3^+^, CD68^+^, CD5^+^/CD19^+^, CD19^+^) of PBMCs from CLL patients (*n* = 3) and healthy donors (*n* = 3). PBMCs were unstimulated or stimulated with PMA/ionomycin (PMA) in presence of brefeldin A (BFA). Dot plots are representative examples of TL1A flow cytometry analysis in gated-cell subsets (CD3^+^, CD68^+^, CD5^+^/CD19^+^, CD19^+^) of PBMCs from CLL patient and healthy donor. Number in bold indicate the percentage of TL1A-positive cells.

To identify the possible cell source of TL1A, first we analyzed specific TL1A mRNA expression in different cell subsets (CD3^+^, CD14^+^, CD19^+^) isolated by FACS from PBMCs of CLL patients and healthy donors. As shown in Figure 5A, TL1A gene was expressed in each analyzed subset showing a great variability among samples, with some samples exhibiting a clear positive TL1A expression and others showing TL1A relative expression values ≤ 1, which represents the mean TL1A mRNA across samples.

Next, we analyzed TL1A protein expression in the T-cell (CD3^+^), monocyte (CD68^+^), and B-cell (CD5^+^/CD19^+^ for CLL and CD19^+^ for healthy donors) subsets of CLL and healthy-donor PBMCs, using intracellular staining and flow cytometry in the presence of cytokine secretion inhibition (BFA). The CD68 pan-monocyte staining was used instead of the CD14 as PMA used in the next experimental step down-regulates CD14 expression. Consistent with data on TL1A-mRNA, within each analyzed cell subset from both CLL and healthy donors, the fraction of TL1A-expressing cells varied across samples, with some samples having a relevant percentage of positive cells and others showing no positive cells with respect to the control (Figure 5B). Interestingly, in TL1A-positive samples, TL1A appeared to be coordinately expressed, both in CLL and healthy donors (Figure 5B and data not shown).

To determine whether TL1A expression was inducible, CLL and healthy-donor PMBCs were stimulated with PMA, in the presence of cytokine secretion inhibition (BFA). As shown in Figure 5B, PMA was able to increase or *de novo* induce TL1A protein in CD68^+^ cells of all the analyzed samples derived from CLL as well as healthy donors. Treatment with PMA induced increased TL1A levels or no change in the CD3^+^ and CD19^+^ subsets of healthy-donor PBMCs (Figure 5B). In contrast, PMA induced no change or even down-regulated TL1A expression within the CLL CD3^+^ and CD5^+^/CD19^+^ cell subsets in CLL samples (Figure 5B). The presence of DR3 and its ligand TL1A in CLL led us to explore the possible function of TL1A in CLL.

### Exogenous TL1A reduces CLL cell proliferation without affecting cell survival

**Figure 6:**
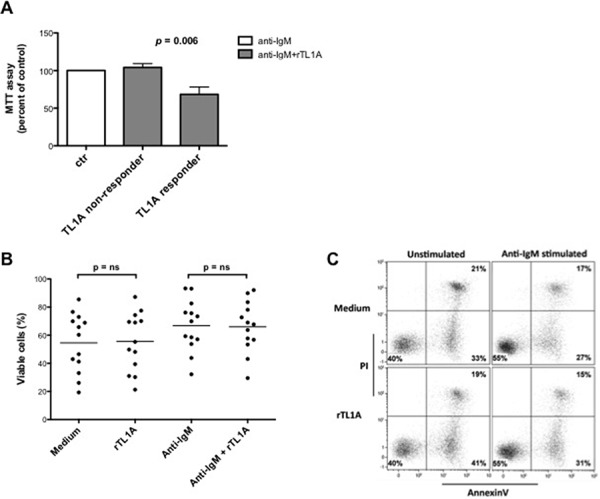
TL1A reduces CLL proliferation induced by stimulation of BCR with anti-IgM without affecting cell survival **A.** CLL samples (*n* = 13) were stimulated with anti-IgM 5 μg/ml in the presence or absence of 100 ng/ml rTL1A for 96 h, and proliferation was analyzed using MTT assay. Proliferation in the presence of anti-IgM was referred as 100%. Data are represented as mean percentage ± SEM. Comparison between the TL1A-responder (*n* = 4) and TL1A-non-responder (*n* = 9) samples was performed using the Kruskal-Wallis test. **B.** Survival was analyzed by Annexin V/PI flow cytometry assay. The same CLL samples analyzed for proliferation (*n* = 13) were stimulated under the same conditions used for MTT assay, stained with Annexin V/PI and then analyzed by flow cytometry. Analyses were gated on CD5^+^/CD19^+^ cells. Comparison between treatments was performed using the Mann-Whitney test. ns = not significant. **C.** Figure shows a representative example of Annexin V/PI flow cytometry analysis.

Previous studies had shown that TL1A/DR3 interactions *in vitro* enhance cell proliferation of activated T cells [11, 27–28]. On the contrary, we have recently documented an *in vitro* inhibitory function for TL1A on B-cell proliferation [6]. To investigate whether TL1A could modulate cell proliferation in CLL, purified B cells from CLL patients were incubated with exogenous, recombinant TL1A in the presence of anti-IgM stimulation, and cell proliferation was measured using MTT assay. Stimulation of leukemic cells with anti-IgM induced a variable proliferation response among the analyzed cells, the fold-increase in cell number ranging from 1.2 to 4.5 (data not shown). Moreover, under these conditions, all the analyzed CLL cell samples but one expressed detectable amounts of surface DR3. To distinguish differential responses to TL1A, a greater than 20% decrease or increase response was selected as an arbitrary cut-off. As shown in Figure 6A, TL1A reduced proliferation in a fraction of CLL cell samples (TL1A-responder) whereas induced no change in the remaining samples (TL1A-non-responder). All TL1A-responder samples expressed surface DR3 whereas the DR3-negative sample was in the non-responder group. No effect was observed when CLL cells were incubated with TL1A alone (data not shown). Remarkably, responsiveness to TL1A co-stimulation was more frequently detected in samples at an early stage disease at diagnosis [3 of the 4 responder samples (75%) *versus* 4 of the 9 non-responder samples (44%) were Rai 0 at diagnosis], although the association between responsiveness to TL1A and Rai 0 stage was not statistically significant (data not shown). No association was detected between TL1A responsiveness and Binet staging, the status of standard prognostic markers (*IGHV*, CD38 or ZAP-70), or cytogenetics (data not shown).

Depending on the cellular context in which DR3 is triggered, its activation can result in apoptosis induction [29]. This raised the possibility that reduced MTT values induced in CLL cells by TL1A were due to reduced cell survival. To test this, purified CLL-B cells were incubated with exogenous recombinant TL1A in the presence of anti-IgM, and cell survival was measured with Annexin V/PI staining and flow cytometry. As shown in Figure 6B and 6C, TL1A did not influence either the spontaneous apoptosis process or the protective effect exerted by anti-IgM, thus demonstrating that reduced MTT values induced by TL1A in anti-IgM-activated CLL cells was due to decreased proliferation and not to reduced survival.

## DISCUSSION

This study documents that the TL1A/DR3 molecular system is expressed in CLL and functions as a negative regulator of leukemic cell proliferation. Over the last decade, a number of evidence has traced a function of broad-acting T-cell stimulator for TL1A/DR3, particularly in inflammatory and autoimmune diseases [15, 30–31]. The key advance of this study is identifying for the first time a B-cell negative regulatory function for the TL1A/DR3 system in a leukemic context. This study assesses the association between TL1A/DR3 expression and early stage disease and points out the importance of the TL1A/DR3 axis in the physiopathology of CLL.

DR3 expression is restricted to lymphocyte-enriched tissues and is especially upregulated in activated T cells [3]. B cells express a combination of DR3 mRNA isoforms [3] and we have recently described that B cells activated by the BCR stimulation express DR3 protein [6]. In this study, we show that leukemic B cells from CLL patients express surface DR3 in response to BCR stimulation. This result has been confirmed in CLL lymph nodes showing that DR3 may be expressed by leukemic cells, presumably activated *in vivo* by the antigen encounter. Indeed, in CLL antigenic stimulation is the most prominent pathogenic mechanism within the lymph-node microenvironment [23–25] and the signaling pathways triggered by the antigen encounter might also drive DR3 expression. In future studies, it would be of interest to identify the signaling key nodes involved in DR3 upregulation. Remarkably, DR3 is more frequently expressed in activated leukemic cells from CLL patients at an early clinical stage at diagnosis, suggesting a role for DR3 at this stage of disease. The relevance of DR3 expression in CLL is confirmed by the presence, in the sera from CLL patients, of its unique ligand TL1A that, similarly to DR3 expression, is more frequently detected in CLL patients at an early-stage disease.

Importantly, exogenous recombinant TL1A reduces cell proliferation in a fraction of CLL cells activated by BCR stimulation. This result is consistent with our recent data showing that TL1A reduces proliferation of healthy B cells activated by BCR stimulation and IL-2 [6]. In contrast, in T cells the TL1A/DR3 axis has been described to act as a costimulator that promotes cell proliferation [11, 27–28]. Overall, these data confirm that either stimulatory or inhibitory regulatory functions may be ascribed to the TL1A/DR3 axis, apparently depending on cellular and/or microenvironmental contexts.

The physiopathology of CLL is entwined with its microenvironment and over the last decade major efforts have been made to characterize driving molecular events that confer to CLL microenvironments peculiarity of permissive niches for cell proliferation and survival [19]. Within these microenvironments, a crucial role may be also played by inhibitory mechanisms of cell proliferation, which may alter the balance between cell death and proliferation favoring the clearance of leukemic cells. Accordingly, the differential expression of these mechanisms may have a direct impact on patients’ clinical outcome. Herein, we show that in some CLL patients and healthy donors, T cells, monocytes, and B cells are able to produce TL1A, apparently in a coordinate fashion. Our data are consistent with previous studies showing that monocytes and T cells are able to produce TL1A [11, 32] whereas they document for the first time the ability of leukemic and healthy B cells to express TL1A. Further studies will be required to identify the specific TL1A-expressing cell subsets within the T-cell, monocyte, and B-cell populations.

Within the lymph nodes, interactions with T cells promote survival of CLL cells [33] whereas the monocyte-derived nurse-like cells support the survival and proliferation of CLL cells [34–36]. Besides this supportive role, our study suggests that T cells and monocytes may also play a regulatory role on CLL growth, presumably at an early-stage disease. Moreover, the finding that leukemic B cells may produce TL1A and express DR3 suggests the existence of an autocrine mechanism acting to regulate leukemic growth. Also, we show that similar regulatory mechanisms may be active in a healthy context.

Our study documents that TL1A may be upregulated following treatment with PMA in each analyzed cell subset from healthy donors and in the monocyte subset from CLL, consistent with previous data showing that TL1A mRNA is induced by PMA in HUVECs [7]. In contrast, in T cells and leukemic B cells from CLL patients, PMA induces no change or even TL1A downregulation. This behavior may be ascribed to functional defects and features of exhaustion documented in T cells from patients with CLL [37–39] whilst, in leukemic B cells, it may be due to functional alterations compatible with the anergic state described in the majority of CLL cells [40–43].

Herein, we document that expression as well as functional traits of TL1A/DR3 axis are predominantly observed in patients with an early disease specifically designated by the Rai 0 stage, irrespectively of the clinical stage as defined by the Binet classification system. Indeed, at variance with the Binet three-stage classification system, the Rai staging designates five clinical stages, thus allowing the identification of a low-risk, early-stage group of patients who are not captured by classification based on the Binet staging or the molecular prognostic parameters. Moreover, we have recently reported that the TL1A/DR3 phenotypic and functional trait is also expressed in non-leukemic B cells, where it may have regulatory functions [6]. These findings lead us to hypothesize that in CLL the TL1A/DR3 functional axis may be preserved after the leukemic transformation, at least until the early stages of disease, where it may contribute to negatively regulate leukemic cell proliferation. In contrast, the TL1A/DR3 functional trait appears to be absent or impaired in advanced stages of CLL, thus representing an escape mechanism from the control of cell proliferation. Interestingly, our results are consistent with a study from Poggi et al. [44], which shows that lack of the inhibitory receptor LAIR-1 (leukocyte-associated Ig-like receptor 1) is associated with high-risk CLL. Moreover, it has been recently described that lack of LAIR-1 expression is able to predict CLL disease progression [45].

In conclusion, we propose a novel regulatory function for TL1A/DR3 that, differentially expressed in relation with clinical stage, may alter the balance between cell proliferation and death, influencing CLL physiopathology and clinical outcome. In addition to its pathogenic relevance, the presence of the TL1A/DR3 system might represent a molecular marker for a finer classification of patients with early-stage disease.

## MATERIALS AND METHODS

### Cell and tissue samples

Peripheral blood mononuclear cells (PBMC) from 36 CLL patients and 11 age-matched healthy donors were collected and cryopreserved at the Hematology Unit, Azienda Ospedaliera Universitaria Integrata (AOUI) in Verona (Italy); CLL tissue lymph-node specimens were collected at the Pathology Unit, AOUI. The local Ethics Committee (Comitato Etico per la Sperimentazione, AOUI) approved sample collection. In accordance with the Declaration of Helsinki, all patients provided written informed consent for the collection and use of their samples for research purposes. Patient inclusion criteria were the lack of prior treatment for at least 6 months prior to sample collection and availability of clinical annotation.

Diagnosis was based on 1996 National Cancer Institute-Working Group/IWCLL and 2008 Guidelines for Diagnosis and Treatment of CLL [46, 47]. CLL patient characteristics at diagnosis are summarized in Table 1. Patients were stratified into major cytogenetic categories, based on NCCN CLL Guidelines: favorable (13q as a sole aberration), neutral (normal karyotype, 12q trisomy), and unfavorable (11q and/or 17p deletion).

**Table 1:** Characteristics of CLL patients

	*n* (%)
***n***	36
**Age**	
Mean	64
Range	32–88
**Gender**	
F	16 (44%)
M	20 (56%)
**Rai stage**	
0	15 (42%)
I	11 (31%)
II	7 (19%)
III	0 (0%)
IV	3 (8%)
**Binet stage**	
A	28 (78%)
B	5 (14%)
C	3 (8%)
***IGHV* status**^1^	
Mutated	17 (47%)
Unmutated	19 (53%)
**CD38**^2^	
Negative	19 (53%)
Positive	17 (47%)
**ZAP-70**^2^	
Negative	18 (50%)
Positive	18 (50%)
**Cytogenetics**^3^	
Neutral	16 (44%)
Favorable	7 (19%)
Unfavorable	10 (28%)
n.a.	3 (8%)

1 *IGHV* sequencing utilized a 2% cut-off to discriminate mutated from unmutated *IGHV;*

2 Percent ZAP-70- and CD38-positive cells were determined by setting the threshold at 20% and 30%, respectively [49]

3 Cytogenetic analysis was performed using fluorescence *in situ* hybridization (FISH) [50–51]

n.a. = not available.

For DR3 surface expression, Western blot and proliferation experiments, B cells were isolated by negative selection using Human B-Cell Enrichment Kit, without CD43 depletion (Stem Cell Technologies Stem Cell Technologies, Vancouver, Canada).

### Flow cytometry analysis

For analysis of DR3 surface expression, purified B cells from 36 patients and 10 healthy-donors were stimulated with sulfate latex beads (Interfacial Dynamics Corporation, Portland, OR, USA) [48] coated with anti-IgM (cat#2022-01, SouthernBiotech, Birmingham, AL, USA). Purified B cells incubated with uncoated beads were used as control. Then, cells were harvested and stained with anti-DR3-PE (clone JD3, cat#307106, BioLegend, San Diego, CA, USA) or isotype-matched control antibody; anti-CD19-APC; anti-CD5-PE-Cy7 and 7-amino-actinomycin (7-AAD) viable dye (all BD Biosciences, Franklin Lakes, NJ, USA).

For analysis of intracellular TL1A expression, PBMCs from 3 patients and 3 healthy-donors were cultured with 10 μg/ml brefeldin A (BFA) in presence or absence of 25 ng/ml phorbolmyristate (PMA), 1 μg/ml ionomycin, for 6 hours. Cells were fixed and permeabilized with Fix&Perm^®^ Kit (Nordic-MUbio, Susteren, Netherlands) and incubated with either anti-TL1A mAb (clone L4G9, cat#ALX-804-832-C100, Enzo Life Sciences, Farmingdale, NY, USA) or isotype-matched control antibody, followed by staining with an anti-mouse-PE secondary antibody (Dako, Glostrup, Denmark). Then, cells were incubated with anti-CD19-BV510, anti-CD5-V450, anti-CD-56-PE-Cy7, anti-cPARP-AlexaFluor647 (all BD Biosciences), anti-CD68-AlexaFluor488, and anti-CD3-APC-Cy7 (both BioLegend).

Samples were acquired on a FACSCanto II cytometer (Becton Dickinson, San Jose, CA, USA). DR3 data were analyzed using FlowJo software (TreeStar, Ashland, OR, USA); TL1A data were analyzed using Kaluza software (Beckman Coulter, Miami, FL, USA). Median fluorescence intensity (MFI) was normalized with respect to MFI of control antibody. Fold change in DR3 expression was calculated as the ratio between normalized signals (RMFI) of anti-IgM-stimulated and unstimulated cells.

### Western blot analysis

Purified CLL-B cells from 4 patients, stimulated with anti-IgM-coated beads as for DR3 flow cytometry analysis, were lysed and 15 μg proteins were separated on a SDS-polyacrylamide gel and Western blotting was performed according to standard procedures, using anti-DR3 antibody (cat# 3254, Cell Signaling Technology, Danvers, MA, USA) for detection. Signals were quantified with ImageJ software (http://rsb.info.nih.gov/ij/) and normalized calculating the ratio between DR3 and β-actin signals. DR3 fold change was calculated as the ratio between normalized signals of anti-IgM stimulated and unstimulated cells.

### Lymph-node immunofluorescence

Immunofluorescence was performed on formalin-fixed paraffin-embedded tissue lymph-node sections from 2 patients, as previously described [6]. Briefly, DR3 expression was detected using anti-DR3-biotinylated mAb (clone JD3, cat#NB100–77847, Novus Biologicals, Cambridge, England). B cells were detected with anti-CD23 and anti-mouse biotinylated secondary antibody (Leica Biosystems, Newcastle, England). Signals were revealed with Qdot Streptavidin Conjugates (QD565, QD655; Invitrogen, Eugene, OR, USA) and Qnuclear Deep Red Stain (Invitrogen). Slides were mounted with Qdot Qmount mounting media (Invitrogen). Images were acquired on a BX61 microscope (Olympus Optical, Tokyo, Japan) and elaborated with CellF software (Olympus Soft Imaging Solutions, Munster, Germany).

### Enzyme-linked immunosorbent assay (ELISA)

Serum levels of TL1A were measured in 26 patients and 11 healthy donors using human TL1A ELISA kit (Alexis-Biochemical, San Diego, CA, USA), according to the manufacturer's protocol. Absorbance was measured using VictorX4 plate reader (Perkin-Elmer, Monza, Italy). All samples were run in duplicate.

### Cell sorting

PBMCs from 6 patients and 3 healthy donors were stained with anti-CD3-FITC, anti-CD19-PE (both BD Biosciences), anti-CD14-APC-Cy7 (BioLegend), and 7-AAD. Stained cells were analyzed and sorted on a FACSAria II instrument (Becton Dickinson), using FACSDiva software (Becton Dickinson). T cells, monocytes, and B cells were identified respectively on the basis of CD3, CD14, and CD19 expression. Purity of FACS-isolated cells was greater than 98%, as assessed by post-sorting analysis.

### Quantitative RT-PCR (qRT-PCR)

Total RNA was extracted from FACS-isolated CD3^+^, CD14^+^ and CD19^+^ cells from patients and healthy donors using TRIzol Reagent (Invitrogen), according to the manufacturer's protocol. RNA was reverse transcribed and qRT-PCR carried out on ABI PRISM 7900HT SDS instrument (Applied Biosystems, Warrington, England). *TNFSF15* (TL1A) was evaluated using Taqman assay Hs00353710_s1 (Applied Biosystems). TL1A expression was calculated by relative quantification, using *ACTB* (β-actin) expression as endogenous reference. Data were analyzed as indicated in User Bulletin #2 (Applied Biosystems). All samples were run in duplicate.

### Proliferation assay

Cell proliferation was measured in 13 CLL samples using MTT (thiazolyl blue; Sigma-Aldrich, Milan, Italy), according to the manufacturer's protocol. Briefly, purified CLL-B cells were stimulated with 5 μg/ml soluble anti-IgM in presence or absence of 100 ng/ml human recombinant TL1A (Peprotech, London, England) for 96 hours, followed by 4 hour-incubation with MTT. Absorbance was measured using VictorX4 plate reader (Perkin-Elmer). All samples were run in duplicate.

### Apoptosis assay

Apoptosis was detected in 13 CLL samples using Annexin V-FITC Apoptosis Detection kit (Bender MedSystem, Vienna, Austria), according to the manufacturer's protocol. Briefly, purified CLL-B cells were stimulated with 5 μg/ml soluble anti-IgM in the presence or absence of 100 ng/ml human recombinant TL1A for 96 hours, followed by staining with Annexin V-FITC and PI and acquisition on a FACSCanto instrument (Becton Dickinson). Data were analyzed using Kaluza software (Beckman Coulter). All samples were run in duplicate.

### Statistical analysis

Graphing and statistical analyses were performed using GraphPad Prism software (GraphPad Software Inc., La Jolla, CA, USA). Results obtained in independent experiments were expressed as mean ± SEM. As data were not normally distributed, the two-sample Wilcoxon signed-rank sum test was used to compare DR3 expression between unstimulated and anti-IgM-stimulated cells. Kruskal-Wallis test was used to compare proliferation data and DR3 and TL1A expression between the cytogenetic risk categories. Mann-Whitney test was used in all other experiments. Differences between the data were considered significant for *p*-values < 0.05.
